# Psychological symptoms and related risk factors among healthcare workers and medical students during the early phase of the COVID‐19 pandemic in Japan

**DOI:** 10.1002/pcn5.5

**Published:** 2022-03-14

**Authors:** Takaki Tanifuji, Shinsuke Aoyama, Yutaka Shinko, Kentaro Mouri, Saehyeon Kim, Seimi Satomi‐Kobayashi, Masakazu Shinohara, Seiji Kawano, Ichiro Sora

**Affiliations:** ^1^ Department of Psychiatry Kobe University Graduate School of Medicine Kobe Japan; ^2^ Department of Medical Education Kobe University Graduate School of Medicine Kobe Japan; ^3^ Department of Epidemiology Kobe University Graduate School of Medicine Kobe Japan

**Keywords:** COVID‐19 pandemic, depression, healthcare worker, medical student, mental health

## Abstract

**Aim:**

The aim of this study was to investigate the mental health status of healthcare workers and medical students during the early phase of the COVID‐19 pandemic.

**Methods:**

An online questionnaire was administered to 637 students and 3189 healthcare workers from May to July, 2020. The patient healthcare questionnaire‐9 (PHQ‐9) and state anxiety (A‐State) of the state–trait anxiety inventory‐form (STAI) were used to assess depression and anxiety symptoms, respectively. Individuals were categorized into severe (15 or higher) depression and severe (50–51 or higher) anxiety groups.

**Results:**

Healthcare workers and those taking care of COVID‐19 patients had a higher risk of severe depression (PHQ‐9 scores >15) than other comparison groups. Students and men also had a higher risk of severe anxiety (STAI > 50–51). Multivariable logistic regression analysis showed that healthcare workers had a fivefold higher risk of developing severe depression symptoms (adjusted odds ratio [OR] = 4.99, confidence interval [CI] 2.24–5.97, *P*‐value < 0.001) and those taking care of COVID‐19 patients had 2.8‐fold higher risk of developing severe depression symptoms (OR 2.75, CI 1.36–5.53, *P*‐value = 0.005).

**Conclusion:**

Both medical students and healthcare workers have been experiencing depression and anxiety symptoms during the first wave of the pandemic. Our findings showed a high rate of severe anxiety symptoms in medical students and a high rate of severe depression symptoms in healthcare workers. Those who treated COVID‐19 patients were at greater risk of developing major depressive disorder than those who treated non‐COVID‐19 patients.

## Graphical abstract

This study investigated the mental health status of healthcare workers and medical students during the early phase of the COVID‐19 pandemic. A high rate of severe anxiety symptoms in medical students and a high rate of severe depression symptoms in healthcare workers were found. Those who treated COVID‐19 patients were at greater risk of developing major depressive disorder than those who treated non‐COVID‐19 patients.

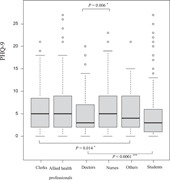

## INTRODUCTION

Since the middle of December 2019, the world has been facing a novel infectious disease known as novel coronavirus disease 2019 (COVID‐19). The Chinese city of Wuhan first reported the outbreak of COVID‐19, which then spread nationally and globally.^1^ By February 2020, there were 17,205 confirmed cases in China and 146 confirmed cases in 23 other countries.^2^ Early research investigated the immediate psychological response to the COVID‐19 epidemic among the general population in China, which revealed that 53.8% of individuals reported the psychological impact of the outbreak as moderate or severe, 16.5% reported moderate or severe depressive symptoms, and 28.8% reported moderate or severe anxiety symptoms.^3^ Pandemics often impact both physical and mental health.^4^, ^5^ According to the World Health Organization's latest research on common mental health disorders, during the COVID‐19 pandemic the prevalence of depression increased by more than three times and that of anxiety increased by more than four times the prevalence in the non‐COVID‐19 period.^6^ In a study including more than 10,000 people in Japan, psychological stress tended to be high among healthcare workers, individuals with pre‐existing mental illness, young people, students, and women during the COVID‐19 pandemic.^7^ The extreme fear and uncertainty experienced during COVID‐19 caused public mental health issues, such as distress reactions, health risk behaviors, and increased occurrence of mental health disorders. It is critical that mental health professionals support vulnerable populations, such as infected patients and healthcare workers, and individuals with pre‐existing conditions.^2^

Previous studies have reported that the incidence of depression and anxiety among healthcare workers was approximately 51% and 41%–45%, respectively, during the COVID‐19 pandemic.^8^, ^9^ Students, who are also a vulnerable population, had no choice but to depend on their parents financially, were unable to go to school, remained worried for their future, undertook online classes, and were isolated.^10^ In the United States, 48.14% of the students reported moderate or high depression and 38.48% reported moderate or high anxiety.^11^ Recently, similar findings on healthcare workers and students have been reported in Japan.^12^, ^13^

Although comparable studies have been carried out globally, the results of these studies vary greatly depending on the background of the people. We need to collect evidence to fully comprehend the psychological impact of the COVID‐19 pandemic in Japan, and appropriately address mental health matters. Hence, the current study aimed to investigate the mental health state of those with depression and anxiety, and identify risk factors related to these symptoms among healthcare workers and medical students during the COVID‐19 pandemic in Japan.

## METHODS

### Study design and participants

This study had a cross‐sectional design and used a survey administered online to 637 students at Kobe University School of Medicine from May 25, 2020 to June 3, 2020, and to 3189 healthcare workers of Kobe University Hospital from June 26, 2020 to July 30, 2020. Students had been absent from school during the survey period due to declaration of a state of emergency. They were not involved in clinical education and did not treat patients with COVID‐19. All students responded to the survey. We received a total of 893 responses from healthcare workers (28%), of which 169 responses were omitted because of omissions or refusal, resulting in 724 total results (22.7%). Demographic data were collected from all participants. For students, we collected data regarding grade level, age, and sex, and for healthcare workers, age, sex, occupation, work environment, and length of service were included. In the work environment, we classified those who tended to patients directly as “contact workers” and the others as “no contact workers.” We defined the work environment of those who treated patients with COVID‐19 (including doubtful ones) as “high‐risk workers” and the others as “low‐risk workers.” An online questionnaire was used to collect participants' demographic data, including their occupation and work environment. Allied health professionals included pharmacists, occupational therapists, physical therapists, speech therapists, orthoptists, clinical psychologists, radiological technologists, medical technologists, clinical engineering technologists, registered dietitians, and dental hygienists. Others included nursing assistants, janitors, and part‐time workers. This survey used the patient healthcare questionnaire‐9 (PHQ‐9)^14^, ^15^ and the state anxiety (A‐State) of the state‐trait anxiety inventory‐form X (STAI‐X)^16^, ^17^ to assess depression and anxiety symptoms, respectively.

### Measures

#### PHQ‐9

The PHQ‐9 is a self‐rating scale for evaluating depression symptoms and comprises nine items rated on a scale from 0 (not at all) to 3 (nearly every day).^14^, ^15^ The validity as well as the reliability of the PHQ‐9 were assessed in a previous study involving the Japanese population. It has widely been used in both clinical and research settings.^18^ The total scores can range from 0 to 27 points; severity is classified into five categories: minimal (0–4), mild,^5^, ^6^, ^7^, ^8^, ^9^ moderate,^10^, ^11^, ^12^, ^13^, ^14^ moderately severe,^15^, ^16^, ^17^, ^18^, ^19^ and severe.^20^, ^21^, ^22^, ^23^, ^24^, ^25^, ^26^, ^27^ In this study, we redefined the depressive groups categories as follows: individuals were categorized into normal (0–9), moderate,^10^, ^11^, ^12^, ^13^, ^14^ and severe (15 or higher) depression groups. The reason for this revision is that PHQ‐9 has been clinically verified, reporting a sensitivity of 88%–92.5% and a specificity of 77%–88% at a cutoff score of 10 or higher (PHQ‐9 ≥ 10), and a sensitivity of 68%–68.8% and a specificity of 91.1%–95% at a cutoff score of 15 or higher (PHQ‐9 ≥ 15).^14^, ^15^

#### STAI

The STAI is a self‐rating scale used for evaluating anxiety symptoms and the validity and reliability of the Japanese version were confirmed under both clinical and research conditions.^16^, ^17^ It is divided into the state anxiety (A‐state) scale and the trait anxiety (A‐trait) scale, each of which consists of 20 items rated on a scale from 1 (not at all) to 4 (exactly). A‐state evaluates current feelings of tension, anxiety, and nervousness and A‐trait refers to individual differences in anxious temperament. We used only STAI‐X. The Japanese STAI‐X rating is as follows: total scores from 20 to 80 points; five sex‐dependent categories of severity: very low (20–22), low (23–31), normal (32–40), high (41–49), and very high (50–80) for men; very low (20–21), low (22–30), normal (31–41), high (42–50), and very high (51–80) for women. We redefined the anxiety groups into three categories as follows: normal (20–40), moderate (41–49), and severe (50 or higher) for men; normal (20–41), moderate (42–50), and severe (51 or higher) for women. In this paper, we will refer to STAI‐X as STAI.

### Statistical analysis

Data were analyzed using the R version 4.0.0 (R development core team, Vienna, Austria) with the EZR version 1.42 (Saitama Medical Center, Jichi Medical University). The *α* was set at 0.05, and data were reported as medians with interquartile ranges (IQRs). The Mann–Whitney *U* test and the Kruskal–Wallis test were used to express the PHQ‐9 and STAI scores. The data of PHQ‐9 and STAI severity were expressed as numbers and percentages, and subjected to Fisher's exact test. The odds ratios (OR) and 95% confidence intervals (CI) were used to reveal the risk factors related to symptoms of depression or anxiety. Multiple logistic regression analysis was also performed after adjustment for relevant factors (^a^adjusted for sex, age; ^b^adjusted for sex, age, length, treating patients directly, treating COVID‐19 patients, and occupation [excluding students], Table 3).

## RESULTS

### Demographic data

In this study, 1361 participants (724 healthcare workers and 637 students) were included. The characteristics of the participants are shown in Table 1. Among healthcare workers, 121 (16.7%) were doctors, 248 (34.3%) were nurses, 130 (18.0%) were allied health professionals, 192 (26.5%) were clerks, and 33 (4.6%) were others. Most healthcare workers were women (532, 73.5%), 20–49 years old (634, 83%), contact workers (519, 71.7%), and low‐risk workers (577, 79.7%). The number of high‐risk workers among contact workers was 147 of 519 (28%). Most students were men (394, 61.9%) and mainly 20–29 years old (484, 76%).

**Table 1:** Demographic characteristics of healthcare workers and medical students

	*N* (%), median (IQR)							
	Occupation							
	Total	Doctors	Nurses	Allied health professionals^a^	Clerks	Others^b^	Healthcare worker totals	Students
Number	1361	121 (16.7)	248 (34.3)	130 (18.0)	192 (26.5)	33 (4.6)	724 (100)	637
Sex								
Women	775 (56.9)	39 (32.2)	224 (90.3)	85 (65.4)	162 (84.4)	22 (66.7)	532 (73.5)	243 (38.1)
Men	586 (43.1)	82 (67.8)	24 (9.7)	45 (34.6)	30 (15.6)	11 (33.3)	192 (26.5)	394 (61.9)
Generation								
<0	137 (10.1)	0	0	0	0	0	0	137 (21.5)
20–29	655 (48.1)	26 (21.5)	80 (32.3)	38 (29.2)	24 (12.5)	3 (9.1)	171 (23.6)	484 (76.0)
30–39	249 (18.3)	49 (40.5)	84 (33.9)	40 (30.8)	54 (28.1)	7 (21.2)	234 (32.3)	15 (2.4)
40–49	200 (14.7)	32 (26.4)	52 (21.0)	35 (26.9)	68 (35.4)	12 (36.4)	199 (27.5)	1 (0.2)
50–59	109 (8.0)	10 (8.3)	30 (12.1)	16 (12.3)	43 (22.4)	10 (30.3)	109 (15.1)	0
>60	11 (0.8)	4 (3.3)	2 (0.8)	1 (0.8)	3 (1.6)	1 (3.0)	11 (1.5)	0
Length	6.0 (2.0–12.0)	5.0 (2.0–11.0)	10.0 (4.0–16.0)	6.0 (2.0–11.0)	3.0 (1.0–7.0)	4.0 (1.0–12.0)	6.0 (2.0–12.0)	‐
Treating patients directly^c^								
Contact workers	519 (71.7)	114 (94.2)	227 (91.5)	108 (83.1)	128 (66.7)	6 (18.2)	519 (71.7)	‐
No‐contact workers	205 (28.3)	7 (5.8)	21 (8.5)	22 (16.9)	64 (33.3)	27 (81.8)	205 (28.3)	‐
Treating COVID‐19 patients^d^								
High‐risk workers	147 (20.3)	34 (28.1)	85 (34.3)	24 (18.5)	3 (1.6)	1 (3.0)	147 (20.3)	‐
Low‐risk workers	577 (79.7)	87 (71.9)	163 (65.7)	106 (81.5)	189 (98.4)	32 (97)	577 (79.7)	‐

Abbreviations: *N*, number of people; IQR, interquartile range; PHQ‐9, patient healthcare questionnaire‐9; STAI, state trait anxiety inventory.

^a^ Occupational therapists, physical therapists, speech therapists, orthoptists, clinical psychologists, radiological technologists, medical technologists, clinical engineering technologists, registered dietitians, dental hygienists.

^b^ Nursing assistants, janitors, part‐time workers.

^c^ We defined those who attended to patients directly as “contact workers” and the others as “no‐contact workers.”

^d^ We defined those who took care of patients with COVID‐19, including suspected cases, as “high‐risk workers” and the others as “low‐risk workers.”

### Severity of anxiety and depression

Of all participants, 662 (48.7%) had moderate to severe symptoms of anxiety and 203 (14.9%) had moderate to severe symptoms of depression. In particular, among healthcare workers 282 (39.0%) had moderate to severe symptoms of anxiety and 150 (20.7%) had moderate to severe symptoms of depression, while among students 380 (59.7%) had moderate to severe symptoms of anxiety and 53 (8.3%) had moderate to severe symptoms of depression. Furthermore, among healthcare workers 18 (2.5%) had severe symptoms of anxiety and 52 (7.2%) had severe symptoms of depression, while among students 54 (8.5%) had severe symptoms of anxiety and 12 (1.9%) had severe symptoms of depression (Table 2).

**Table 2:** Severity and scores of depression and anxiety in healthcare workers and medical students

	*N* (%), median (IQR)									
	Occupation									
	Total	Doctors	Nurses	Allied health professionals^a^	Clerks	Others^b^	*P*‐value	Healthcare worker totals	Students	*P*‐value
STAI	41.0 (37.0–44.0)	40.0 (37.0–44.0)	40.0 (36.0–42.0)	39.0 (36.0–43.0)	40.0 (36.0–42.25)	42.0 (40.0–44.0)	0.08	40.0 (36.0–43.0)	42.0 (39.0–46.0)	<0.0001***
Normal	699 (51.4)	70 (57.9)	154 (62.1)	80 (61.5)	124 (64.6)	14 (42.4)		442 (61.0)	257 (40.3)	{<0.0001}***
Moderate	590 (43.4)	45 (37.2)	91 (36.7)	45 (34.6)	66 (34.4)	17 (51.5)		264 (36.5)	326 (51.2)	
Severe	72 (5.3)	6 (5.0)	3 (1.2)	5 (3.8)	2 (1.0)	2 (6.1)		18 (2.5)	54 (8.5)	
PHQ‐9	4.0 (2.0–7.0)	3.0 (2.0–7.0)	5.0 (2.0–9.0)	5.0 (2.0–8.75)	5.0 (2.0–8.25)	4.0 (2.0–9.0)	0.014*	5.0 (2.0–9.0)	3.0 (1.0–6.0)	<0.0001 ***
Normal	1158 (85.1)	103 (85.1)	191 (77)	105 (80.8)	149 (77.6)	26 (78.8)		574 (79.3)	584 (91.7)	{<0.0001}***
Moderate	139 (10.2)	14 (11.6)	39 (15.7)	11 (8.5)	29 (15.1)	5 (15.2)		98 (13.5)	41 (6.4)	
Severe	64 (4.7)	4 (3.3)	18 (7.3)	14 (10.8)	14 (7.3)	2 (6.1)		52 (7.2)	12 (1.9)	

We evaluated *P*‐value with the Mann–Whitney *U* test or the Kruskal–Wallis test for continuous variables such as scores of the PHQ‐9 and STAI.

We evaluated *P*‐value with the Fisher's exact test for categorical variables such as classified data of PHQ‐9 and STAI severity.

Abbreviations: *N*, number of people; IQR, interquartile range; PHQ‐9, patient health care questionnaire‐9; STAI, state trait anxiety inventory.

^a^ Occupational therapists, physical therapists, speech therapists, orthoptists, clinical psychologists, radiological technologists, medical technologists, clinical engineering technologists, registered dietitians, dental hygienists.

^b^ Nursing assistants, janitors, part‐time workers.

{}, *P*‐value including all severity categories.

*P‐value < 0.05; **P‐value < 0.001; ***P‐value < 0.0001.

### Psychiatric symptoms and associated factors

**Figure 1:**
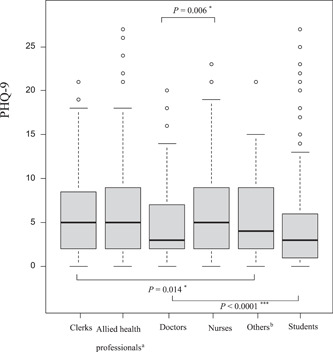
Comparisons of depression symptoms with the PHQ‐9 in healthcare workers and medical students. The median (IQR) scores on the PHQ‐9 for depression among healthcare workers showed significant differences (*P*‐value = 0.014). The post hoc multicomparison tests indicated that nurses scored significantly higher than doctors (*P*‐value = 0.006). The comparison between healthcare workers and students showed a significant difference (*P*‐value < 0.001). ^a^Occupational therapists, physical therapists, speech therapists, orthoptists, clinical psychologists, radiological technologists, medical technologists, clinical engineering technologists, registered dietitians, dental hygienists. ^b^Nursing assistants, janitors, part‐time workers. *P‐value < 0.05; **P‐value  0.001; ***P‐value < 0.0001.

The median (IQR) STAI scores for anxiety among doctors, nurses, allied health professionals, clerks, and others were 40 (37–44), 40 (36–42), 39 (36–43), 40 (36–42.25), and 42 (40–44), respectively (*P*‐value = 0.08). The median STAI scores for all healthcare workers and students were 40 (36–43) and 42 (39–46), respectively (*P*‐value < 0.001; Table 2 and Figure S1). The median PHQ‐9 scores for doctors, nurses, allied health professionals, clerks and others were 3.0 (2.0–7.0), 5.0 (2.0–9.0), 5.0 (2.0–8.75), 5.0 (2.0–8.25), and 4.0 (2.0–9.0), respectively (*P*‐value = 0.014). On post hoc multicomparison tests, nurses scored significantly higher than doctors (*P*‐value = 0.006). The median PHQ‐9 scores for all healthcare workers and students were 5.0 (2.0–9.0) and 3.0 (1.0–6.0), respectively (*P*‐value < 0.001; Table 2 and Figure 1). While the median scores on both questionnaires did not show a significant grade‐related difference for students, they showed a significant age‐related difference for healthcare workers, which was not significantly different on post hoc multicomparison tests (Tables S1 and S2); younger healthcare workers tended to have high PHQ‐9 scores (Table S1). Men had higher scores and more severe ratios on the STAI than women (among healthcare workers, men vs. women: median [IQR] = 41 [38–44] vs. 39 [36–42], *P*‐value = 0.001; severe ratio of the STAI, among healthcare workers, men vs. women, *n* [%] = 11 [5.7%] vs. 7 [1.3%], *P*‐value < 0.0001; among students, men vs. women, *n* [%] = 46 [11.7%] vs. 8 [3.3%], *P*‐value < 0.001; Table S3). Women healthcare workers and students had higher PHQ‐9 scores than men (among healthcare workers, women vs. men: median [IQR] = 5.0 [2.0–9.0] vs. 4.0 [2.0–8.0], *P*‐value = 0.017; among students, women vs. men: median [IQR] = 4.0 [1.0–6.0] vs. 3.0 [1.0–6.0], *P*‐value = 0.024; Table S3). There were no significant differences between contact workers and no contact workers in either questionnaire data. However, high‐risk workers had higher scores and more severe ratios on the PHQ‐9 than those of low‐risk workers (among high‐risk workers vs. low‐risk workers: median [IQR] = 5.0 [2.0–10.0] vs. 5.0 [2.0–8.0], *P*‐value = 0.025; severe ratio of the PHQ‐9, high‐risk workers vs. low‐risk workers, *n* [%] = 19 [12.9%] vs. 33 [5.7%], *P*‐value = 0.016; Table S4).

### Risk factors associated with severity of anxiety and depression

Multivariate logistic regression analysis showed that after controlling for confounding factors, men were more likely to present with moderate to severe anxiety than women for both healthcare workers and students groups (moderate to severe, among healthcare workers: adjusted OR [CI] = 2.47 [1.66–3.68], *P*‐value = < 0.0001; among medical students: adjusted OR [CI] = 1.40 [1.01–1.94], *P*‐value = 0.043; severe, among healthcare workers: adjusted OR [CI] = 3.26 [1.08–9.83], *P*‐value = 0.036; among medical students: adjusted OR [CI] = 3.82 [1.77–8.24], *P*‐value < 0.001; Table 3). Healthcare workers had lower odds of developing anxiety symptoms than students, who had approximately 1.4‐fold higher odds of having moderate to severe anxiety symptoms (adjusted OR = 1.43, *P*‐value = 0.036; Table 3). Healthcare workers were approximately five times more likely to have severe depression symptoms (adjusted OR [CI] = 4.99 [2.24–5.97], *P*‐value = < 0.001; Table 3). Compared with low‐risk workers, high‐risk workers had approximately 2.8 times increased odds of having severe depression symptoms (adjusted OR [CI] = 2.75 [1.36–5.53], *P*‐value = 0.005; Table 3). Multicollinearity was accurate in these analyses. The correlations between PHQ‐9 and STAI scores of all participants, healthcare workers, and students were determined by Spearman's test correlation: −0.0832, *P*‐value = 0.0021; 0.0412, *P*‐value = 0.268; and −0.119, *P*‐value = 0.0027, respectively (Table S5).

**Table 3:** Risk factors associated severity of depression and anxiety in healthcare workers and medical students

	OR	95% CI	*P*‐value	Adjusted OR	95% CI	*P*‐value
STAI, moderate–severe anxiety symptoms						
Healthcare workers (vs. students)	**0.43**	**0.35**–**0.54**	**<0.0001** ***	**0.70** a	**0.50**–**0.98**	**0.036** *
Male healthcare workers (vs. female)	**2.22**	**1.58**–**3.10**	**<0.0001** ***	**2.47** b	**1.66**–**3.68**	{**<0.0001**}***
Male students (vs. female)	**1.39**	**1.00**–**1.92**	**0.047** *	**1.40** a	**1.01**–**1.94**	**0.043** *
Contact workers treating patients directly (vs. no‐contact workers)^c^	0.97	0.70–1.35	0.845	0.87^b^	0.55–1.36	0.529
High‐risk workers treating COVID‐19 patients (vs. low‐risk workers)^d^	1.32	0.91–1.90	0.143	1.20^b^	0.80–1.80	0.387
STAI, severe anxiety symptoms						
Healthcare workers (vs. students)	**0.28**	**0.16**–**0.48**	**<0.0001** ***	0.42^a^	0.17–1.04	0.061
Male healthcare workers (vs. female)	**4.56**	**1.74**–**11.90**	**0.002** *	**3.26** b	**1.08**–**9.83**	**0.036** *
Male students (vs. female)	**3.88**	**1.80**–**8.38**	**<0.0001** ***	**3.82** a	**1.77**–**8.24**	**<0.001** **
Contact workers treating patients directly (vs. no‐contact workers)^c^	1.03	0.36–2.92	0.959	0.69^b^	0.15–3.14	0.627
High‐risk workers treating COVID‐19 patients (vs. low‐risk workers)^d^	1.12	0.37–3.47	0.838	0.91^b^	0.267–3.12	0.883
PHQ‐9, moderate–severe depression symptoms						
Healthcare workers (vs. students)	**2.88**	**2.06**–**4.02**	**<0.0001** ***	**3.80** a	**2.42**–**5.97**	{**<0.0001**}***
Male healthcare workers (vs. female)	0.85	0.56–1.28	0.433	0.92^b^	0.57–1.49	0.737
Male students (vs. female)	1.22	0.67–2.20	0.513	1.20^a^	0.67–2.18	0.539
Contact workers treating patients directly (vs. no‐contact workers)^c^	1.06	0.71–1.59	0.764	1.10^b^	0.65–1.89	0.717
High‐risk workers treating COVID‐19 patients (vs. low‐risk workers)^d^	1.45	0.95–2.21	0.086	1.56^b^	0.98–2.51	0.063
PHQ‐9, severe depression symptoms						
Healthcare workers (vs. students)	**4.03**	**2.13**–**7.62**	**<0.0001** ***	**4.99** a	**2.24**–**11.10**	{**<0.0001**}***
Male healthcare workers (vs. female)	1.13	0.61–2.12	0.693	1.14^b^	0.55–2.39	0.723
Male students (vs. female)	1.24	0.37–4.16	0.729	1.21^a^	0.36–4.08	0.757
Contact workers treating patients directly (vs. no‐contact workers)^c^	1.72	0.85–3.49	0.135	2.12^b^	0.87–5.17	0.100
High‐risk workers treating COVID‐19 patients (vs. low‐risk workers)^d^	**2.49**	**1.35**–**4.44**	**0.003** *	**2.75** b	**1.36**–**5.53**	**0.005** *

*Note*: The boldface indicates a significant difference.

Abbreviations: *N*, number of people; IQR, interquartile range; OR, odds ratio; CI, confidence interval; PHQ‐9, patient health care questionnaire‐9; STAI, state trait anxiety inventory.

^a^ Adjusted for sex and age.

^b^ Adjusted for sex, age, length, contact with patients; contact with COVID‐19 patients, occupation (excluding student).

^c^ We defined those who attended to patients directly as “contact workers” and the others as “no‐contact workers.”

^d^ We defined those who taking care of patients with COVID‐19, including suspected cases, as “high‐risk workers” and the others as “low‐risk workers.”

{}, *P*‐value including all severity categories.

*P‐value < 0.05; **P‐value < 0.001; ***P‐value < 0.0001.

## DISCUSSION

This study aimed to assess the psychiatric symptoms of healthcare workers and medical students during the early stage of the COVID‐19 pandemic in Japan. A total of 1361 participants, with 724 healthcare workers and 637 students, were enrolled. Our results indicated that healthcare workers, nurses, women, younger people, and high‐risk workers were likely to have depression symptoms, while students and men had a higher risk of severe anxiety. Those taking care of COVID‐19 patients had an approximately threefold higher risk of developing severe depression than those who treated non‐COVID‐19 patients. This is in line with previous findings that high psychological stress affected healthcare workers, young people, and women during the COVID‐19 pandemic in Japan.^7^ Another study on healthcare workers reported similar trends, showing that women, young people, and those in the frontline were likely to have high Depression Anxiety and Stress Scales 21 (DASS‐21) scores.^8^

Medical students had high ratios of moderate to severe anxiety symptoms and an approximately 1.4‐fold risk of developing them, whereas healthcare workers had a lower‐ratio. Although the COVID‐19 pandemic had just commenced, healthcare workers may have experienced less anxiety because of their general medical knowledge. Wang et al. indicated that most students had anxiety related to academic progress, academic performance, career path after graduation, inability to go to school, isolation, and self‐restraint.^11^ Recent studies of the COVID‐19 pandemic indicated that the anxiety symptoms were risk factors of depressive symptoms.^13^, ^19^ In consistency with the previous findings, our study showed that anxiety symptoms of students were the prodrome to depressive symptoms, indicated by the fact that the number of students committing suicides after May 2020 was very high compared to those in other years.^20^, ^21^ However, healthcare workers were not the case and might have felt more tired and depressive than anxious because it was after the prodrome because exhaustion is related with fatigue and loss of energy in major depressive disorder, which results in a depressive emotional state. However, healthcare workers might have felt more tired and depressive than anxious because it was after the prodrome.^22^

Although many studies showed that anxiety symptoms are more common in women than in men,^23^, ^24^, ^25^, ^26^, ^27^ our study showed that men were at a greater risk of developing severe anxiety symptoms. While psychiatric symptoms during the COVID‐19 pandemic varied among countries,^25^ several studies conducted during the COVID‐19 pandemic in Asia using the generalized anxiety disorder seven‐item scale (GAD‐7) support the findings of our study, which is based on the STAI.^28^, ^29^ Longitudinal study during the COVID‐19 pandemic in Japan showed that from April to July for the population below 40 years the PHQ‐9 scores increased almost equally and GAD‐7 scores increased more steeply in men than in women.^21^ Most of our study participants were below 40 years, and our study may reflect similar results.

Many studies have reported that women and younger people are vulnerable populations in pandemics such as COVID‐19 and H1N1.^4^, ^5^, ^30^ Our results showed that younger healthcare workers and women tended to have strong depression symptoms. Some epidemiological studies suggest that women are at a higher risk of developing depression and anxiety, pointing to gender inequality as one potential reason.^23^, ^24^, ^31^ In Japan, 92% of nurses are women,^32^ and our results also showed that nurses had the highest PHQ‐9 scores among healthcare workers. This may be because nurses and younger healthcare workers generally have many opportunities to care for patients directly, and have to engage in a wide range of stress‐inducing tasks.

Of the 724 healthcare workers, 52 (7.2%), and of the 637 students, 12 (1.9%) had severe symptoms of depression. The Japanese version of PHQ‐9 has been clinically verified with a sensitivity of 68.8%, specificity of 91.1%, and likelihood ratio of 7.7% at a cutoff score of 15 or higher.^15^ Although depression symptoms are heterogeneous and psychiatric examination is needed for diagnosis, people scoring higher than 15 may have a high risk of developing major depressive disorder. In our study, just under 10% of the healthcare workers scored higher than 15. Of the healthcare workers 150 (20.7%) had moderate to severe symptoms of depression, and of the students 53 (8.3%) had moderate to severe symptoms of depression. Based on previous studies involving the general Chinese and Japanese populations using the PHQ‐9, 736 (12%) of the 6130 participants experienced moderate to severe symptoms of depression in China (PHQ‐9 score > 9),^28^ and 17.3% of 2000 and 18.35% of 2708 participants experienced moderate to severe symptoms of depression in Japan (PHQ‐9 score > 9).^29^, ^33^ Healthcare workers were more likely to have moderate to severe symptoms of depression than medical students. If we applied the cutoff score of 10 or higher, as in many studies, one in five healthcare workers would be considered to have a high risk of developing depression.^4^, ^9^, ^15^, ^28^ In our experimental design, medical students could be considered as a substitute for non‐healthcare workers because a previous study reported that the psychiatric symptoms are roughly comparable between students and other occupations.^34^ Healthcare workers had approximately five times the risk of developing severe depression symptoms compared to non‐healthcare workers. Although according to a previous study, high‐risk workers were not at an increased risk of developing depression,^13^ those who treated patients with COVID‐19 (including suspected cases) had approximately three times the risk of developing severe depression symptoms.^5^, ^9^, ^35^ In contrast, comparison between contact workers and no‐contact workers showed no significant differences.

Since resources are limited, it is necessary that mental healthcare interventions should be carried out efficiently based on the results of studies like ours. We must try to recognize risk factors for mental illness, acknowledge each other's dedicated work through positive messages, discuss psychological and physical health, and openly share concerns so that susceptibility to developing depression symptoms can be minimized.^8^, ^36^

This study has several limitations. First, it was limited by its cross‐sectional design and lacked longitudinal follow‐up, so it does not reflect the long‐term effect of the pandemic. Additionally, data collection duration and timing were different between healthcare workers and students. Second, this study used self‐report questionnaires, which rely on subjectivity and are somewhat unreliable. Third, the target population was limited and this may have made our study biased. It is also possible that healthcare workers and medical students have more medical knowledge than general population. As for non‐healthcare workers, we could only collect information from students. Fourth, a small number of healthcare workers responded (28% response rate) because of omissions or refusal to participate in research, therefore the final response rate was 22.7%. Given these considerations, our results may be biased. Finally, we could not assess the detailed characteristics of the participants, such as life events or stressors. Indeed, we wanted to further investigate our interesting findings that the severity of depression symptoms was not correlated with anxiety symptoms, and men were at a greater risk of developing severe anxiety symptoms than women, but we were not able to do this because of lack of information.

## CONCLUSIONS

We evaluated the psychiatric symptoms of healthcare workers and medical students during the first wave of the COVID‐19 pandemic in Japan. The individuals who treated patients with COVID‐19, healthcare workers, nurses, women, and younger people were vulnerable to depression symptoms. Medical students and men were vulnerable to severe anxiety symptoms. It is possible that healthcare workers were more likely to develop severe depressive symptoms, especially as those who treated COVID‐19 patients had a higher risk of developing severe depression.

## CONFLICTS OF INTEREST

The authors declare no conflicts of interest.

## ETHICS APPROVAL STATEMENT

Our study design and all related procedures were performed in accordance with the Declaration of Helsinki. This study was approved by the Ethical Committee of Kobe University Graduate School of Medicine (Approval number B200155). We made secondary use of the results of a survey conducted for public health. All participants were given the opportunity to refuse enrolling to our study and their consent was obtained securely.

## AUTHOR CONTRIBUTIONS

Shinsuke Aoyama designed the research procedure. Takaki Tanifuji was responsible for analyzing the data and drafting the manuscript. Yutaka Shinko and Saehyeon Kim analyzed the data. Seimi Satomi‐Kobayashi, Masakazu Shinohara, Saehyeon Kim collected the data. Shinsuke Aoyama, Kentaro Mouri and Ichiro Sora revised the manuscript. All authors read and approved the final submission.

## Supporting information

Supporting information.

Supporting information.

## Data Availability

The raw data supporting the conclusions of this article are subject to the following restrictions: data usage for secondary purpose is required to be approved by the Ethical Committee of Kobe University Graduate School of Medicine. Requests to access these datasets should be directed to Shinsuke Aoyama, aoyama@med.kobe-u.ac.jp.
