# Supplementary material for: Psychological symptoms and related risk factors among healthcare workers and medical students during the early phase of the COVID‐19 pandemic in Japan
Source: PCN Rep. 2022 Mar 14;1(1):e5. doi: 10.1002/pcn5.5 (PMC9088491; doi:10.1002/pcn5.5)
Supplement: Supplementary file 1 — Supporting information. [file PCN5-1-e5-s002.docx]

***Supporting information materials***

**Psychological symptoms and related risk factors among health care workers and medical students during the early phase of COVID-19 pandemic in Japan**

Takaki Tanifuji^1^, Shinsuke Aoyama^1^*, Yutaka Shinko^1^, Kentaro Mouri^1^, Saehyeon Kim^1^, Seimi Satomi-Kobayashi^2^, Masakazu Shinohara^3^, Seiji Kawano^2^, Ichiro Sora^1^

^1^Department of Psychiatry, Kobe University Graduate School of Medicine, Kobe, Japan

^2^ Department of Medical Education, Kobe University Graduate School of Medicine, Kobe, Japan

^3^ Department of Epidemiology, Kobe University Graduate School of Medicine, Kobe, Japan

**Correspondence:**Corresponding Author: Shinsuke Aoyama, M.D, PhD
Email: [aoyama@med.kobe-u.ac.jp](mailto:aoyama@med.kobe-u.ac.jp)

**Supporting information Table 1.**

|  | N (%), Median (IQR) | |  |  |  |  |
| --- | --- | --- | --- | --- | --- | --- |
|  | Generation of health care workers | | |  |  |  |
|  | 20-29 | 30-39 | 40-49 | 50-59 | >60 | *p-*value |
| STAI | 41.0 (38.0-44.0) | 40.0 (36.0-42.75) | 40.0 (37.0-42.0) | 39.0 (36.0-42.0) | 45.0 (35.5-47.5) | 0.012 |
| Normal | 92 (53.8) | 139 (59.4) | 131 (65.8) | 75 (68.8) | 5 (45.5) |  |
| Moderate | 74 (43.3) | 87 (37.2) | 65 (32.7) | 33 (30.3) | 5 (45.5) |  |
| Severe | 5 (2.9) | 8 (3.4) | 3 (1.5) | 1 (0.9) | 1 (9.1) |  |
| PHQ-9 | 6.0 (3.0-  9.0) | 5.0 (2.0-9.0) | 4.0 (2.0-8.0) | 4.0 (2.0-7.0) | 2.0 (1.0-6.0) | 0.039 |
| Normal | 131 (76.6) | 179 (76.5) | 163 (81.9) | 91 (83.5) | 10 (90.9) |  |
| Moderate | 27 (15.8) | 35 (15.0) | 24 (12.1) | 11 (10.1) | 1 (9.1) |  |
| Severe | 13 (7.6) | 20 (8.5) | 12 (6.0) | 7 (6.4) | 0 (0.0) |  |

**Supporting information Table 2.**

|  | N (%), Median (IQR) | |  |  |  |  |  |
| --- | --- | --- | --- | --- | --- | --- | --- |
|  | Grade of students | |  |  |  |  |  |
|  | 1sth | 2nd | 3rd | 4th | 5th | 6th | *p-*value |
| Number | 110 (17.3) | 110 (17.3) | 108 (17) | 100 (15) | 111 (17.4) | 98 (15.4) |  |
| STAI | 42.5 (39.0-46.0) | 42.0 (39.0-47.75) | 42.5 (40.0-46.0) | 43.0 (39.0-47.0) | 41.0 (37.5-46.0) | 42.0 (39.0-46.0) | 0.598 |
| Normal | 42 (38.2) | 47 (42.7) | 34 (31.5) | 40 (40.0) | 53 (47.7) | 41 (41.8) |  |
| Moderate | 64 (58.2) | 50 (45.5) | 68 (63.0) | 49 (49.0) | 50 (45.0) | 45 (45.9) |  |
| Severe | 4 (3.6) | 13 (11.8) | 6 (5.6) | 11 (11.0) | 8 (7.2) | 12 (12.2) |  |
| PHQ-9 | 3.0 (1.0-  6.0) | 3.0 (1.0-6.0) | 3.0 (1.0-5.0) | 3.0 (1.0-6.0) | 3.0 (1.0-5.0) | 3.5 (1.0-7.0) | 0.568 |
| Normal | 103 (93.6) | 101 (91.8) | 102 (94.4) | 88 (88.0) | 106 (95.5) | 84 (85.7) |  |
| Moderate | 5 (4.5) | 6 (5.5) | 6 (5.6) | 11 (11.0) | 3 (2.7) | 10 (10.2) |  |
| Severe | 2 (1.8) | 3 (2.7) | 0 (0.0) | 1 (1.0) | 2 (1.8) | 4 (4.1) |  |

**Supporting information Table 3.**

|  | N (%), Median (IQR) | |  |  |  |  |
| --- | --- | --- | --- | --- | --- | --- |
|  | Sex of health care workers | | | Sex of students | |  |
|  | Men | Women | *p*-value | Men | Women | *p*-value |
| STAI | 41.0 (38.0-44.0) | 39.0 (36.0-42.0) | 0.001 ^*^ | 42.0 (39.0-47.0) | 42.0 (39.0-46.0) | 0.463 |
| Normal | 90 (46.9) | 352 (66.2) | {<0.0001 ^***^} | 147 (37.3) | 110 (45.3) | {<0.001 ^**^} |
| Moderate | 91 (47.4) | 173 (32.5) |  | 201 (51.0) | 125 (51.4) |  |
| Severe | 11 (5.7) | 7 (1.3) |  | 46 (11.7) | 8 (3.3) |  |
| PHQ-9 | 4.0 (2.0-  8.0) | 5.0 (2.0-9.0) | 0.017 *^*^* | 3.0 (1.0-6.0) | 4.0 (1.0-6.0) | 0.024 ^*^ |
| Normal | 156 (81.2) | 418 (78.5) | {0.459} | 359 (91.1) | 225 (92.6) | {0.830} |
| Moderate | 21 (10.9) | 77 (14.5) |  | 27 (6.9) | 14 (5.8) |  |
| Severe | 15 (7.8) | 37 (7.0) |  | 8 (2.0) | 4 (1.6) |  |

**Supporting information Table 4.**

|  | N (%), Median (IQR) | |  |  |  |  |
| --- | --- | --- | --- | --- | --- | --- |
|  | Treating patients directly ^a^ | |  | Treating COVID -19 patients ^b^ | | |
|  | Contact workers | No contact  workers | *p-*value | High-risk | Low-risk | *p-*value |
| STAI | 40.0 (36.0-  43.0) | 40.0 (37.0-  43.0) | 0.356 | 40.0 (37.0-43.0) | 40.0 (36.0-43.0) | 0.127 |
| Normal | 318 (61.3) | 124 (60.5) | {0.971} | 82 (55.8) | 360 (62.4) | {0.331} |
| Moderate | 188 (36.2) | 76 (37.1) |  | 61 (41.5) | 203 (35.2) |  |
| Severe | 13 (2.5) | 5 (2.4) |  | 4 (2.7) | 14 (2.4) |  |
| PHQ-9 | 5.0 (2.0-  9.0) | 5.0 (2.0-  9.0) | 0.616 | 5.0 (2.0-10.0) | 5.0 (2.0-8.0) | 0.025 ^*^ |
| Normal | 410 (79.0) | 164 (80.0) | {0.274} | 109 (74.1) | 465 (80.6) | {0.016 ^*^} |
| Moderate | 67 (12.9) | 31 (15.1) |  | 19 (12.9) | 79 (13.7) |  |
| Severe | 42 (8.1) | 10 (4.9) |  | 19 (12.9) | 33 (5.7) |  |

**Supporting information Table 5.**

|  | correlation coefficient between STAI and PHQ-9 | *p-*value |
| --- | --- | --- |
| All participants | -0.0832 | 0.0021 ^*^ |
| Health care workers | 0.0412 | 0.268 |
| Students | -0.119 | 0.0027 ^*^ |

Clerks

Doctors

Nurses

Others

Students

20

30

40

50

60

70

STAI

*P* = 0.08

*P* < 0.0001

**Supporting information Figure 1.**

Allied health

professionals
