# Supplementary material for: Psychological symptoms and related risk factors among healthcare workers and medical students during the early phase of the COVID‐19 pandemic in Japan
Source: PCN Rep. 2022 Mar 14;1(1):e5. doi: 10.1002/pcn5.5 (PMC9088491; doi:10.1002/pcn5.5)
Supplement: Supplementary file 2 — Supporting information. [file PCN5-1-e5-s001.docx]

**Supporting information materials legends**

**Supporting information Table 1. Severity and Scores of Depression and Anxiety in Health Care Workers by age group**

We evaluated the p-value with the Kruskal–Wallis test for continuous variables such as scores of the PHQ-9 and STAI.

We evaluated p-value with the Fisher's exact test for categorical variables such as classified data of PHQ-9 and STAI severity.

Abbreviations: N, number of people; IQR, interquartile range; STAI, state trait anxiety inventory; PHQ-9, patient health care questionnaire-9

^*^ p-value < 0.05

^**^ p-value < 0.001

^***^ p-value < 0.0001

**Supporting information Table 2. Severity and Scores of Depression and Anxiety in Medical Students by age group**

We evaluated p-value with the Kruskal–Wallis test for continuous variables such as scores of the PHQ-9 and STAI.

We evaluated p-value with the Fisher's exact test for categorical variables such as classified data of PHQ-9 and STAI severity.

Abbreviations: N, number of people; IQR, interquartile range; STAI, state trait anxiety inventory; PHQ-9, patient health care questionnaire-9.

^*^ p-value < 0.05

^**^ p-value < 0.001

^***^ p-value < 0.0001

**Supporting information Table 3. Severity and Scores of Depression and Anxiety by sex**

We evaluated p-value with the Mann–Whitney U test for continuous variables such as scores of the PHQ-9 and STAI.

We evaluated p-value with the Fisher's exact test for categorical variables such as classified data of PHQ-9 and STAI severity.

Abbreviations: N, number of people; IQR, interquartile range; STAI, state trait anxiety inventory; PHQ-9, patient health care questionnaire-9

{}: p-value including all severity categories

^*^ p-value < 0.05

^**^ p-value < 0.001

^***^ p-value < 0.0001

**Supporting information Table 4. Severity and Scores of Depression and Anxiety in the Workplace**

^a^ We defined those who attended to patients directly as “contact workers” and the other as “no contact workers”.

^b^ We defined those who taking care of patients with COVID-19 including suspected cases as "high-risk workers" and the other as "low-risk workers."

We evaluated p-value with Mann–Whitney U test for continuous variables such as scores of the PHQ-9 and STAI.

We evaluated p-value with the Fisher's exact test for categorical variables such as classified data of PHQ-9 and STAI severity.

Abbreviations: N, number of people; IQR, interquartile range; STAI, state trait anxiety inventory; PHQ-9, patient health care questionnaire-9

{}: p-value including all severity categories

^*^ p-value < 0.05

^**^ p-value < 0.001

^***^ p-value < 0.0001

**Supporting information Table 5. Correlation between Depression and Anxiety in Health Care Workers and Medical Students**

Abbreviations: STAI, state trait anxiety inventory; PHQ-9, patient health care questionnaire-9

^*^ p-value < 0.05

^**^ p-value < 0.001

^***^ p-value < 0.0001

**Supporting information Figure 1. Comparisons of Anxiety Symptoms with the STAI in Health Care Workers and Medical Students**

The median (IQR) scores on the STAI for anxiety among healthcare workers did not show significant difference (p-value = 0.08). The comparison between healthcare workers and students showed significant difference (p-value < 0.001).

^a^ occupational therapist, physical therapist, speech therapist, orthoptist, clinical psychologist, radiological technologist, medical technologist, clinical engineering technologist, registered dietitian, dental hygienist.

^b^ nursing assistants, janitors, part-time workers.

^*^ p-value < 0.05

^**^ p-value < 0.001

^***^ p-value < 0.0001
